# Pain as a global public health priority

**DOI:** 10.1186/1471-2458-11-770

**Published:** 2011-10-06

**Authors:** Daniel S Goldberg, Summer J McGee

**Affiliations:** 1 Department of Bioethics & Interdisciplinary Studies, Brody School of Medicine, East Carolina University, 600 Moye Blvd, Mailstop 641, Greenville, N.C. 27834, USA; 2 Department of Health Policy & Management, University of Kansas School of Medicine/KU Medical Center, Center for Practical Bioethics, The Harzfeld Building, 1111 Main St. Ste. 500 Kansas City, MO 64105, USA

## Abstract

**Background:**

Pain is an enormous problem globally. Estimates suggest that 20% of adults suffer from pain globally and 10% are newly diagnosed with chronic pain each year. Nevertheless, the problem of pain has primarily been regarded as a medical problem, and has been little addressed by the field of public health.

**Discussion:**

Despite the ubiquity of pain, whether acute, chronic or intermittent, public health scholars and practitioners have not addressed this issue as a public health problem. The importance of viewing pain through a public health lens allows one to understand pain as a multifaceted, interdisciplinary problem for which many of the causes are the social determinants of health. Addressing pain as a global public health issue will also aid in priority setting and formulating public health policy to address this problem, which, like most other chronic non-communicable diseases, is growing both in absolute numbers and in its inequitable distribution across the globe.

**Summary:**

The prevalence, incidence, and vast social and health consequences of global pain requires that the public health community give due attention to this issue. Doing so will mean that health care providers and public health professionals will have a more comprehensive understanding of pain and the appropriate public health and social policy responses to this problem.

## Background

By any measure, pain is an enormous global health problem. Globally, it has been estimated that 1 in 5 adults suffer from pain and that another 1 in 10 adults are diagnosed with chronic pain each year [1]. While pain affects all populations, regardless of age, sex, income, race/ethnicity, or geography, it is not distributed equally across the globe. Those who experience pain can experience acute, chronic, or intermittent pain, or a combination of the three. The four largest causes of pain are cancer, osteo- and rheumatoid arthritis, operations and injuries, and spinal problems, making the etiology of pain a complex, transdisciplinary affair. Pain has multiple, serious sequelae including but not limited to depression, inability to work, disrupted social relationships and suicidal thoughts. Of those living with chronic pain, the median time of exposure is 7 years [1].

In this article, we argue that the paradigmatic view of pain as a symptom of disease, rather than as a disease state itself, [2] has contributed to the neglect of this condition in the world of public health. Raising awareness about pain for the public health community requires clearly defining pain as a disease state and demonstrating why it must be a public health priority. The undertreatment of pain, a persistent problem for those who experience it, only can be reduced with better diagnosis and treatement applied from a public health framework. Understanding pain as a disease may reduce the global burden of this health problem and its co-morbid conditions as well as potentially decrease the undertreatment and misdiagnosis of pain. These issues ought to be among the major priorities for public health in the next century. Public health officials must gain a better understanding of the magnitude and characteristics of the problem, contribute to the development and evaluation of pain management programs, and develop the best possible interventions to diagnose and treat chronic pain.

## Discussion

### Priority Setting for Pain and Public Health

The setting of public health priorities is a complex, controversial and political process. These priorities are determined, it has been argued, by those who have wealth, political power or both [3]. In the United States, for example, public health priorities are often set by scientific advisory boards such as the Institute of Medicine committee which produced in July 2011 the report *Relieving Pain in America*[4]. What we see in this landmark report is a reframing of the agenda for pain including a social transformation of pain diagnosis, treatment, and care. Yet even though this report argues that pain ought to be framed as a public health issue, it fails to articulate what ought to be as understood as most important about pain from the perspectives of social justice, health inequities, or even the global burdens and causes of disease.

There are multiple reasons for regarding pain as a public health priority. The first and foremost is the staggering prevalence of pain. Because pain is a multivalent, dynamic, and ambiguous phenomenon, it is notoriously difficult to quantify, and therefore caution is warranted in issuing broad assessments regarding the epidemiology of chronic pain across the globe. Yet, even with such limitations, there is little question regarding its high prevalence and incidence. 10% of the world's population - approximately 60 million people - endure chronic pain,[1] and fairly reliable estimates in individual countries and regions indicate chronic pain prevalence closer to 20-25% [5-7]. Primary care settings in Asia, Africa, Europe, and in the Americas had patients reporting persistent pain prevalence of 10 to 25%. Consistent estimates of chronic pain prevalence in the U.S. range from 12 to 25%, and prevalence of 20% has been noted in Europe [7-9]. Although there are few estimates of the incidence of global chronic pain, WHO has estimated that as many as 1 in 10 adult individuals are newly diagnosed with chronic pain each year [1].

Moreover, aside from the prevalence and incidence of chronic pain, both the severity of such pain and the extent of any accompanying disability are also key factors in assessing its burden. The evidence suggests that moderate-to-severe pain is prevalent even in resource-rich settings, [10,11] and that the combination of persistent pain and comorbid psychological disorders produce significant disability across the globe (as measured by impairment of daily activities) [12].

Ultimately, as one commentator recently concluded, the evidence is strong that "for millions of people across the globe, excruciating pain is an inescapable reality of life." [13] This is true for both the developed and the developing world. Pain is therefore a global health problem, one that significantly affects both the global North and the global South. However, akin to the general global burden of disease, pain and its treatment are distributed highly unequally in both the global North and the global South, with those most disadvantaged bearing higher burdens of persistent pain and lesser likelihood of effective treatment. Such inequities render the equitable treatment of pain an ethical imperative consistent with mandates of social justice. While the ethical implications of the inequitable distribution of global chronic pain is properly regarded as a top priority in global health ethics, a full explication is beyond the scope of the current project. Here, we argue simply that pain ought to be regarded as a global public health priority, a point which establishes a baseline for future ethical analyses.

Second, akin to many other chronic non-communicable diseases (NCDs) across the globe, chronic pain is typically accompanied by substantial comorbidities. The roster of chronic NCDs that are positively correlated with chronic pain are myriad, [14] including but not limited to diabetes, [15,16] arthritis, [17] depression, [18,19] and asthma, [20] among others. Chronic NCDs account for over 60 percent of the global burden of mortality, [21] with generally increasing epidemiologic trajectories across the globe [22,23]. Because these NCDs can be expected to increase the incidence and ultimately the prevalence of global chronic pain, the matter of incidence and the larger context regarding comorbid (chronic) NCDs also justifies the treatment of chronic pain as a global public health priority.

### A Public Health Approach to Pain

Understanding pain as a public health priority helps to explain its tight linkage with social and economic determinants of health. The social epidemiologic evidence suggests strongly that patterns of NCDs from the hyperlocal to the global level are powerfully determined by the conditions in which people and communities live, work, and play [23]. Given the aforementioned comorbid relationship between a variety of NCDs and chronic pain, one would predict that chronic pain should correlate with some of these social determinants of health. This prediction is borne out in research that links with chronic pain a number of such determinants, including mental and physical stress at work, [24,25] socioeconomic status, [26] rurality,[27] occupational status, [28] neighborhood, [29] race, [29,30] and education [31].

One of the most promising theories regarding the causal pathways between such social factors and poor NCD and all-cause health outcomes is the allostatic load hypothesis [32,33]. This theory posits that persistent exposure to deleterious social and economic conditions activates the human body's fight-or-flight response to a state of perpetuity. In turn, the persistent accumulation of stress hormones such as cortisol has been robustly correlated with a number of diseases and negative health outcomes [33]. Under this theoretical framework, one would predict a direct relationship between increased social disadvantage and either or both the frequency and the severity of chronic pain. Several recent analyses bear this prediction out [34-36]. Dorner et al. found that even at the same intensity of pain, those lowest on the social gradient reported feeling two to three times as disabled as compared to the highest group [34]. Similarly, Rios and Zautra documented a significant correlation between the severity of daily pain and levels of daily financial worry [35]. Moreover, the fact that social disadvantages tend to cluster implies that communities suffering from any one such disadvantage are more likely to experience others [37,38]. This clustering results in compound effects of deleterious social and economic conditions on health, which, given the stress-health connection, supports a belief in the pathways between such conditions and pain across the globe.

An enormous body of literature demonstrates quite strongly that policy action on these social determinants of health cannot be left to the health care sector itself [23,39,40]. As important as medical services are to ameliorating disease and human suffering, medical care is neither intended to nor generally equipped to address the macrosocial factors that determine patterns of pain and its distribution in populations. Understanding the powerful connections between deleterious social and economic conditions and global chronic pain therefore provides additional reason for regarding pain as a public health priority rather than simply a medical priority.

Moreover, the primary response to the undertreatment of pain both in the developed world and beyond has closely tracked a medical model, one which analyzes the problem almost exclusively in context of the availability and distribution of opioid analgesics [9,41-43]. Yet as Ichiro Kawachi observed in the award-winning documentary *Unnatural Causes*, however useful aspirin may be in the treatment of fever, the cause of fever is not lack of aspirin [44]. Moreover, while there is no question that access to essential medicines is a global health priority, the Final Report of the WHO's Commission on the Social Determinants of Health makes plain the abundant evidence that remedies for the most pressing and inequitable global health problems are typically to be found outside the provision of health care services [23]. Thus, this essay strikes outside the confines of the medical model, and argues that global chronic pain ought to be perceived as a public health priority.

Finally, regarding chronic pain as a global public health priority could have a salutary effect on chronic pain research. Where the medical model dominates research and funding on pain, research allocation is similarly skewed towards basic science (pain at the molecular level) and to clinical medicine, the latter consisting largely of a focus on medical treatments and health care services. Given the likelihood that many of the key determinants of pain across the globe are the social and economic conditions in which people work and live, regarding chronic pain as a global public health priority could shift the emphasis to funding research directed at the root structural, social and cultural factors that shape pain and its inequitable global distribution.

### Summary

The high prevalence and incidence of global chronic pain, its substantial and growing comorbidities, and its linkage with myriad social and economic determinants collectively provide ample justification for regarding pain as a public health priority. Moreover, there are significant public policy consequences for doing so. Thinking about global chronic pain as a public health priority implies immediately that the global focus on access to essential medicines like opioids is insufficient as a primary policy strategy. A public health focus requires stakeholders to heed Rose's insistence that ministering to the health of populations necessitates attention to the causes of the causes [45,46]. Chronic pain, akin to most diseases on the planet, is strongly determined by the social and economic conditions in which people work and live [47]. However important essential medicines are to treating chronic pain, a public health model recommends that significant global attention and resources be shifted to the macrosocial determinants that most powerfully shape the patterns of chronic pain, its comorbid diseases, and the distribution of both. This shift in policy from the micro-level focus on the provision of health care services to a macro-level approach addressing the structural determinants of health dovetails with a number of practices and reports urging the same kind of health policy shift, including but not limited to the World Health Organization, the Marmot Review, and the Commission to Build a Healthier America.

## Competing interests

Neither DSG nor SJM has any competing interests to declare.

## Authors' contributions

DSG and SJM conceived of the manuscript together and participated in planning the writing, workflow, and timeline. DSG wrote the first draft. SJM reviewed the first draft and offered substantial revisions, which DSG and SJM iteratively incorporated into second and third drafts. All authors read and approved the final manuscript.

## Authors' Information

DSG has been working on the issue of pain and its undertreatment since 2005. The issue was the subject of DSG's 2009 doctoral dissertation, which earned a distinction, and which currently serves as the basis for a book proposal under review at a university press. DSG's general approach to the undertreatment of pain, regarding the error of reducing it to a question of opioid policy, has resulted in a number of publications, commentaries, and presentations in peer-reviewed journals and conferences, as well as invitations to speak at a variety of local, national, and international venues. DSG has also produced multiple publications on global health and public health ethics, and has papers on both subjects forthcoming in major scholarly journals and in an anthology on global health to be published by Oxford University Press. DSG was recently named a 2012 Visiting Fellow with the Birkbeck Pain Project at Birkbeck, University of London.

SJM has published over a dozen papers in the area of public health and ethics. The topic of ethics and pain is the focus of the Center for Practical Bioethics' PAINS Project, for which SJM is a consultant. SJM has multiple forthcoming publications in the area of pain, public health, and ethics and is currently developing a book on the ethics of prevention. As the first public health ethicist at the University of Kansas Medical Center, SJM is developing new courses and research programs in public health, pain, and cancer.

## Pre-publication history

The pre-publication history for this paper can be accessed here:

http://www.biomedcentral.com/1471-2458/11/770/prepub
